# Life-course trajectories of body mass index and subsequent cardiovascular risk among Chinese population

**DOI:** 10.1371/journal.pone.0223778

**Published:** 2019-10-10

**Authors:** Md. Tauhidul Islam, Jette Möller, Xingwu Zhou, Yajun Liang

**Affiliations:** 1 Department of Public Health Sciences, Karolinska Institutet, Stockholm, Sweden; 2 Initiative for Non-Communicable Diseases, Health System and Population Studies Division, icddr,b, Dhaka, Bangladesh; 3 Department of Medical Sciences, Clinical Physiology, Uppsala University, Uppsala, Sweden; National Institute of Health and Nutrition, National Institutes of Biomedical Innovation, Health and Nutrition, JAPAN

## Abstract

**Background:**

Examining body mass index (BMI) change over life course is crucial for cardiovascular health promotion and prevention. So far, there is very few evidence on the long-term change of BMI from childhood to late life. This study aimed to examine the life-course trajectory patterns of BMI and then to link the trajectory patterns to cardiovascular risk factors in adulthood.

**Methods:**

Based on longitudinal data from the China Health and Nutrition Survey, 5276 participants (aged 6–60) at baseline (in 1989) with up to 7 measurements of BMI during 1989–2009 were selected in this study. Cardiovascular risk factors including high blood pressure, high blood glucose and high blood lipids were assessed in 2411 participants in 2009. Latent growth curve modelling was used to analyse the BMI trajectories, and logistic regression was used to examine the associations between trajectory patterns and cardiovascular risk factors.

**Results:**

Four trajectories patterns of BMI over life course (age 6–80) were identified: Normal-Stable (22.4% of the total participants), Low normal-Normal-Stable (44.1%), Low normal-Normal-Overweight (27.2%), and Overweight-Obese (4.3%). Compared to those with Normal-Stable pattern, those with Low normal-Normal-Stable pattern, Low normal-Normal-Overweight pattern and Overweight-Obese pattern had higher risk of high blood pressure (odds ratio range = 1.6–6.6), high blood glucose (1.7–9.1), dyslipidemia (2.6–5.9) and having at least two of the three cardiovascular risk factors (3.9–30.9).

**Conclusions:**

Having a stable BMI within normal range over life course is associated with the lowest cardiovascular risk, whereas remaining overweight and obese over life course is associated with the highest cardiovascular risk.

## Introduction

Globally cardiovascular diseases (CVDs) are the leading cause of death [1,2]. There was a substantial increase (by around 15%) in mortality due to CVDs from 2006 to 2016 [1]. CVDs are also the leading cause of disability in the world accounting for almost 12% of global disability-adjusted life years [3]. Furthermore, most of the CVDs deaths in low income countries occurred in the working aged population (younger than 60 years), thus, the loss of labour income will result in a reduction in gross domestic product [4]. Therefore, reducing the burden of CVDs is a global development imperative due to the harmful economic impact [5]. In China, CVDs are also a big burden by accounting for more than 40% of the all-cause mortality, and two in five adults in China are suffering from CVDs [6]. It is projected that from 2010 to 2030 there will be an upsurge of 21.3 million cardiovascular events and 7.7 million cardiovascular deaths in China [7].

Obesity is a well-establised risk factor for CVDs [8]. The Chicago Heart Association Detection Project in Industry conducted in Chicago-area companies and organizations showed that the odds ratio of dying from coronary heart disease was 4 times higher among obese patients in comparison to those who had high blood pressure or high cholesterol level among the industry population [9]. Many randomized control trials have shown that intentional weight loss due to healthy lifestyle interventions at different life periods (either childhood or adulthood) can help to reduce the risk of CVDs [10,11].

Nowadays, many researchers have focused on the life-course prevention of CVDs through studying the growth trajectories. However, the previous studies on body mass index (BMI) trajectories suffered from a limited follow-up period, e.g., childhood only [12–15], adulthood only [16,17], from childhood to young adulthood [18–21], or from childhood (age 7) to midlife (age 49–50) [22–24]. Very few evidence is available on the long-term life-course change of BMI within one individual from childhood to old age (age ≥60), nor findings are found on the cardiovascular risk associated with the life-long change of BMI. In this longitudinal population-based study, we sought to identify the life-course trajectory patterns of BMI and to determine which trajectory pattern was associated with a higher cardiovascular risk in later life (from adulthood to old age).

## Methods

### Study design and participants

Data from the China Health Nutrition Survey (CHNS) was used for this study. CHNS is an ongoing longitudinal survey which was initiated in 1989 among a sample of the Chinese population (aged ≥2 years) and followed up every 2–4 years. The participants of CHNS were selected from 15 provinces and municipal cities through a multistage, random cluster sampling process. Detailed description of the CHNS has been published previously [25]. For the purpose of this cohort study, a secondary analysis was done based on seven waves of CHNS, e.g., 1989, 1991, 1997, 2000, 2004, 2006 and 2009. The survey in 1993 was excluded due to the lack of information on BMI. A total of 5276 participants aged 6 years and older with a valid BMI measurement in 1989 were included in the analyses, and the number of participants followed in 1991, 1997, 2000, 2004, 2006, and 2009 was 4168, 2708, 2371, 2551, 2569, and 2356, respectively. For the analysis of cardiovascular risk factors, 2411 participants from the wave in 2009 were included because of their available measurements of cardiovascular risk factors.

The CHNS study was approved by the institutional review boards from both the University of North Carolina at Chapel Hill, NC, USA and the China Centre for Disease Control and Prevention, Beijing, China. Written informed consents were provided by all participants prior to the surveys and examinations.

### Data collection and definitions

Before the data collection of CHNS, all the interviewers and examiners were well-trained with 7 days’ training provided by the collaborative teams. The data collection was monitored through site visits by the University of North Carolina at Chapel Hill, the China Center for Disease Control and Prevention as well as the China-Japan Friendship Hospital [26]. In addition, the inter-and intra-person reliability was calculated to assess the results of training.

#### Body mass index

Height (in cm) and weight (in kg) were measured using the portable stadiometer and calibrated beam scale, respectively, with light-weight clothing and no shoes. The measurement error was 0.1 cm for height and 0.1 kg for weight. The average value of two measurements in a single visit was recorded as the final value [25]. Height and weight were measured by well-trained examiners following a standard protocol from the World Health Organization [27].

BMI (kg/m^2^) was calculated as weight divided by squared height. Adult overweight was defined as having a BMI ≥24 kg/m^2^ but <28.0 kg/m^2^, and adult obesity was defined as having a BMI ≥28.0 kg/m^2^ [28]. For children and adolescents (aged 6–17 years old), normal weight was defined as having a BMI <85th age- and sex-specific percentile, overweight was defined as having a BMI ≥85th and <95th age- and sex-specific percentile, and obesity was defined as having a BMI ≥95th age- and sex-specific percentile [29].

#### Cardiovascular risk factors

The cardiovascular risk factors (e.g., blood pressure, blood glucose and blood lipids) were collected by well-trained examiners through physical examination and laboratory tests. Blood pressure was measured at each wave, however, blood glucose and blood lipids were measured only at the last follow-up (in 2009) when participants aged 26–80 years old. In this study, only the cardiovascular risk factors measured in 2009 were included.

Systolic blood pressure and diastolic blood pressure were measured on the right arm, using mercury sphygmomanometers with appropriate cuff sizes. Three measurements were collected after a 10-min seated rest. The mean of the three measurements was used in the analysis [30]. Hypertension was defined as having a systolic blood pressure ≥140 mm Hg and/or diastolic blood pressure ≥90 mm Hg or self-report of currently using antihypertensive medication [31].

In the 2009 survey, blood was collected by venipuncture following a 12-hour overnight fast. Fasting plasma glucose was measured with the GOD–PAP method (Randox Laboratories Ltd., UK) [26]. High blood glucose was defined as fasting plasma glucose ≥7.0 mmol/L or self-reported history of physician diagnosis of type 2 diabetes [32]. Blood lipids, such as total cholesterol (TC), triglycerides (TG), high-density lipoprotein cholesterol (HDL-C) and low-density lipoprotein cholesterol (LDL-C), were measured using glycerol-phosphate oxidase method and the polyethylene glycol (PEG)-modified enzyme method, respectively, by determiner regents (Kyowa Medex Co., Ltd., Tokyo, Japan) [26]. Dyslipidemia was defined as having a TC ≥6.22 mmol/L, or TG ≥2.26 mmol/L, or LDL-C≥4.14 mmol/L, or HDL-C <1.04 mmol/L [33].

#### Covariates

Sociodemographic factors (e.g., age, sex, education and region of living) at baseline and lifestyles factors (e.g., smoking, alcohol, diet and physical activity) at last follow-up were considered as the covariates. Smoking was defined as a positive answer to the question “have you ever smoked cigarettes or pipe?” [30]. High alcohol consumption was defined as drinking alcohol more than 3 times a week [30]. Physical inactivity was defined as having less than the equivalent of 150-min moderate-intensity or 75-min vigorous-intensity aerobic physical activity per week [30]. Based on a 3-day record of household meals, unfavourable diet was defined as having at least one of the three macronutrients out of the US recommendation of dietary reference intake (i.e., 45–65% for carbohydrate, 20–35% for fat and 10–35% for protein) [34].

### Statistical analysis

Latent class growth modelling (LCGM) was used for determining the BMI trajectory patterns, i.e., the distinct subgroups of individuals following a similar pattern of BMI change over time [35]. Because gender may have some effect on BMI trajectory [15], the LCGM was adjusted for gender. The exact number of trajectory patterns was identified based on the value of Bayesian Information Criterion (BIC) and posterior probability of LCGM (S1 File). The best model fit was chosen as the one with a lower BIC and a higher posterior probability.

Specifically, the heterogeneity or the distribution of individual differences in BMI change was summarized by a finite set of unique polynomial functions. Details of the polynomial functions described in supporting information (S1 File). The significance of polynomial terms was used to identify the shape of trajectory patterns (e.g., linear versus quadratic). Since BMI might have a nonlinear pattern, a quadratic age term was also added in the trajectory analysis. In addition, LCGM provides information regarding group membership probabilities which indicate the aggregate size of each trajectory or the number of participants in a given trajectory. Preferably, the group membership probability of each trajectory should be at least 5% [35].

Moreover, the calculated posterior probabilities were used to assign each individual membership to the trajectory pattern that best fit the participant’s BMI change. A highest-probability assignment rule was then used to assign each individual membership to the trajectory to which the participant holds the highest posterior membership probability. Following that, the average posterior probability of group membership for a trajectory was calculated, which represented the internal reliability for each trajectory. The average posterior probabilities of group membership greater than 0.80 was taken into consideration to indicate that the modelled trajectories grouped individuals with similar patterns of change and discriminated between individuals with dissimilar patterns of change [35]. The model parameters of LCGM were shown in the S1 Table.

Furthermore, binary logistic regression was used to assess the association between BMI trajectory patterns and cardiovascular risk factors (i.e., high blood pressure, high blood glucose and dyslipidaemia). In addition, we aggregated the cardiovascular risk factors by counting the number and categorized them into three groups (i.e., 0, 1 and ≥2). Then, multinomial logistic regression was used to assess the association between trajectory patterns and clustered cardiovascular risk factors. Odds ratio and 95% confidence interval were used to describe the associations. Since the cardiovascular risk factors were measured at different life periods for different participants, stratified analysis by age at measurement was performed to further assess the association between BMI trajectory pattern and cardiovascular risk factors.

To assess the effect of missing values of BMI on the trajectories, we did multiple imputation with all of the related variables (e.g., age, gender, living region, education, BMI, blood pressure, blood glucose, blood lipids, smoking, alcohol intake, physical activity and diet) into the imputation model. The multiple imputation was performed 5 times. Then, a LCGM was performed for each imputed dataset by assigning the same number of trajectories with same order of terms as that from the original dataset.

All analyses were performed using IBM SPSS 22 for Windows (IBM SPSS Inc., Chicago, Illinois, USA) and SAS version 9.4 (SAS Institute Inc., Cary, NC, USA). The statistical significance level was set at 0.05.

## Results

### Life-course trajectory patterns of BMI

**Fig 1:**
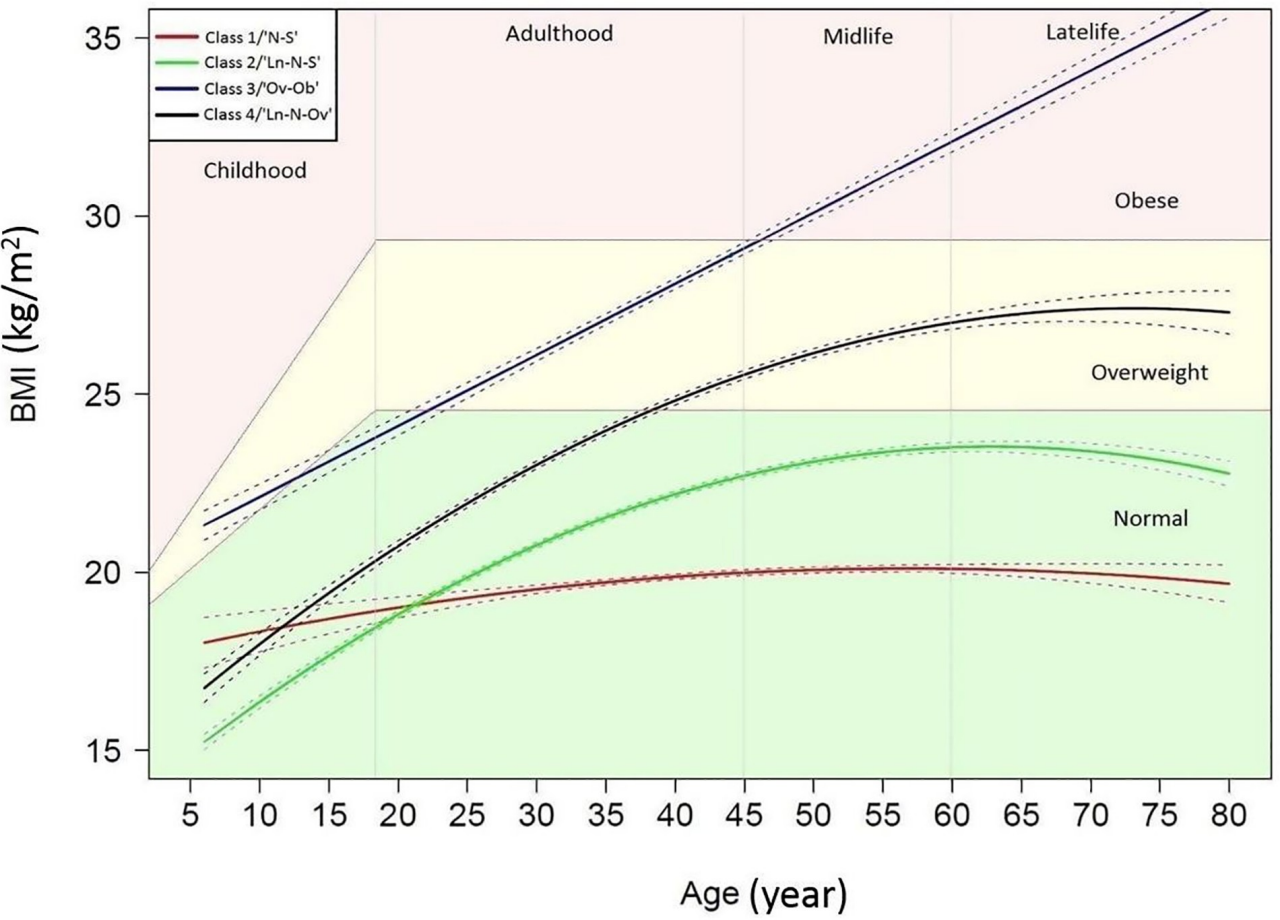
The distinct trajectory patterns of body mass index from childhood to late life. Abbreviations: BMI = body mass index; N-S = Normal- Stable; Ln-N-S = Low normal-Normal- Stable; Ov-Ob = Overweight-Obese; Ln-N-Ov = Low normal-Normal-Overweight. Solid lines represent estimated trajectory patterns. Dashed lines indicate the 95% confidence intervals. Shaded areas represent the BMI status: normal (light green), overweight (light yellow) and obese (light red).

The LCGM identified four trajectory patterns of BMI from 5276 participants (Fig 1). The BIC of the LCGM model was 47650.84 and the average posterior probability ranged from 0.80 to 0.90. 22.4%, 44.1%, 6.3% and 27.2% of the participants were grouped into class 1, class 2, class 3 and class 4, respectively (S1 Table). The four patterns were named as: class 1/Normal-Stable (N-S), class 2/Low normal-Normal-Stable (Ln-N-S), class 3/Overweight-Obese (Ov-Ob), and class 4/Low normal-Normal-Overweight (Ln-N-Ov). The S2 Table showed a detailed description of the naming of each trajectory.

The N-S trajectory pattern showed a stable BMI from childhood (age 6–17), adulthood (age 18–44), midlife (age 45–59) to late life (age ≥60). Ln-N-S trajectory pattern showed a faster increase of BMI from childhood until the age of 60 years and then a slight decrease in late life, whereas the BMI level was always within normal range. Ov-Ob pattern was characterized by a steep increase of BMI over life course, starting from overweight at age 6 to obesity at age 80. Ln-N-Ov pattern also showed a steep increase of BMI, starting from normal BMI in childhood and adulthood to overweight in midlife and late life (Fig 1).

### Characteristics of participants across trajectory patterns

At baseline, in comparison to people with other patterns, participants with N-S pattern had the highest age (*p*<0.001), the proportion of females was highest among those in the Ov-Ob pattern but lowest in those with N-S pattern (*p*<0.001). The N-S pattern had the highest percentage of participants living in a rural area (*p* = 0.007). Participants with Ln-N-Ov trajectory pattern had higher level of education whereas those with N-S pattern had the lowest level of education (*p*<0.001). Participants with the Ov-Ob pattern had the highest level of BMI at baseline (*p*<0.001) (Table 1).

**Table 1:** Characteristics of participants across trajectory patterns at baseline (1989) and last follow-up (2009).

Characteristics^a^	N-S	Ln-N-S	Ln-N-Ov	Ov-Ob	*p*
**Baseline (n = 5276)**					
No of participants	1180	2327	1437	332	
Age, years	34.8 (7.6)	30.3 (9.2)	29.3 (8.3)	29.3 (6.4)	<0.001
Female, n (%)	526 (44.6)	1199 (51.5)	821 (57.1)	221 (66.6)	<0.001
Rural residents, n (%)	849 (71.9)	1600 (68.8)	945 (65.8)	221 (66.6)	0.007
Education, n (%)					
No Education	366 (31.2)	602 (26.5)	313 (22.4)	73 (22.5)	<0.001
Primary School	299 (25.5)	526 (23.1)	300 (21.4)	78 (24.1)	
Middle School and above	509 (43.4)	1147 (50.4)	787 (56.2)	173 (53.4)	
BMI, kg/m^2^	19.5 (1.5)	20.6 (2.2)	22.8 (2.2)	25.7 (2.6)	<0.001
**Last follow-up (n = 2411)**					
No of participants	643	1019	595	154	
Age, years	54.8 (6.8)	53.5 (7.0)	52.0 (7.3)	50.5 (5.4)	<0.001
Female, n (%)	298 (46.3)	534 (52.4)	352 (59.2)	102 (66.2)	<0.001
Rural residents, n (%)	507 (78.8)	770 (75.6)	425 (71.4)	118 (76.6)	0.025
Education, n (%)					
No Education	202 (31.6)	296 (29.1)	157 (26.4)	36 (23.4)	<0.001
Primary School	159 (24.8)	224 (22.0)	113 (19.0)	32 (20.8)	
Middle School and above	279 (43.6)	498 (48.9)	324 (54.5)	86 (55.8)	
BMI, kg/m^2^	20.2 (1.6)	23.4 (1.7)	26.4 (1.9)	29.9 (2.4)	<0.001
Smoking, n (%)	253 (39.3)	327 (32.1)	147 (24.7)	40 (26.0)	<0.001
Alcohol overconsumption, n (%)	111 (17.3)	191 (18.7)	86 (14.5)	17 (11.0)	0.032
Physical inactivity, n (%)	24 (3.7)	43 (4.2)	26 (4.4)	6 (3.9)	0.944
Unfavourable diet, n (%)	410 (64.6)	628 (62.7)	356 (40.4)	96 (62.7)	0.527
SBP, mmHg	121.7 (17.5)	125.2 (18.2)	129.1 (18.0)	133.9 (19.2)	<0.001
DBP, mmHg	78.2 (10.8)	81.1 (11.0)	84.3 (11.1)	88.1 (11.9)	<0.001
Blood glucose, mmol/l	5.2 (1.1)	5.4 (1.3)	5.7 (1.8)	6.2 (2.2)	<0.001
Total cholesterol, mmol/l	4.8 (0.9)	5.0 (1.1)	5.0 (1.0)	5.1 (1.0)	<0.001
LDL-C, mmol/l	2.9 (1.0)	3.1 (1.0)	3.1 (1.0)	3.1 (0.9)	0.002
HDL-C, mmol/l	1.6 (0.5)	1.5 (0.5)	1.4 (0.4)	1.3 (0.5)	<0.001
Triglycerides, mmol/l, median (Q1, Q3)	1.0 (0.7, 1.5)	1.3 (0.9, 1.9)	1.6 (1.1, 2.4)	1.8 (1.2, 3.1)	<0.001

Values are mean (standard deviation), unless otherwise specified. Abbreviations: BMI = body mass index; SBP = systolic blood pressure; DBP = diastolic blood pressure; LDL-C = Low density lipoprotein cholesterol; HDL-C = High density lipoprotein cholesterol; Q1 = first quartile; Q3 = third quartile; N-S = Normal-Stable; Ln-N-S = Low normal-Normal-Stable; Ln-N-Ov = Low normal-Normal-Overweight; Ov-Ob = Overweight-Obese.

^a^The number (%) of missing value was 1 (0.04%) for smoking, 3 (0.1%) for unhealthy drinking and 32 (1.3%) for unhealthy dietary pattern. A dummy variable was created for the missing value and included in subsequent analysis.

At last follow-up, more than 70% of participants in each trajectory pattern lived in a rural area with the highest proportion of rural residents in the N-S trajectory pattern (*p* = 0.025). In comparison with other patterns, the Ov-Ob trajectory pattern had the highest level of education, BMI, systolic blood pressure, diastolic blood pressure, blood glucose, TC, TG, and LDL-C but lowest level of HDL-C (all *p*<0.01), the N-S pattern had the highest prevalence of smoking (*p*<0.001), the Ln-N-S pattern had the highest prevalence of alcohol overconsumption (*p* = 0.032). There was no significant difference in the prevalence of physical inactivity and unfavourable diet among different trajectory patterns (Table 1).

### Cardiovascular risk factors across BMI trajectory patterns

**Fig 2:**
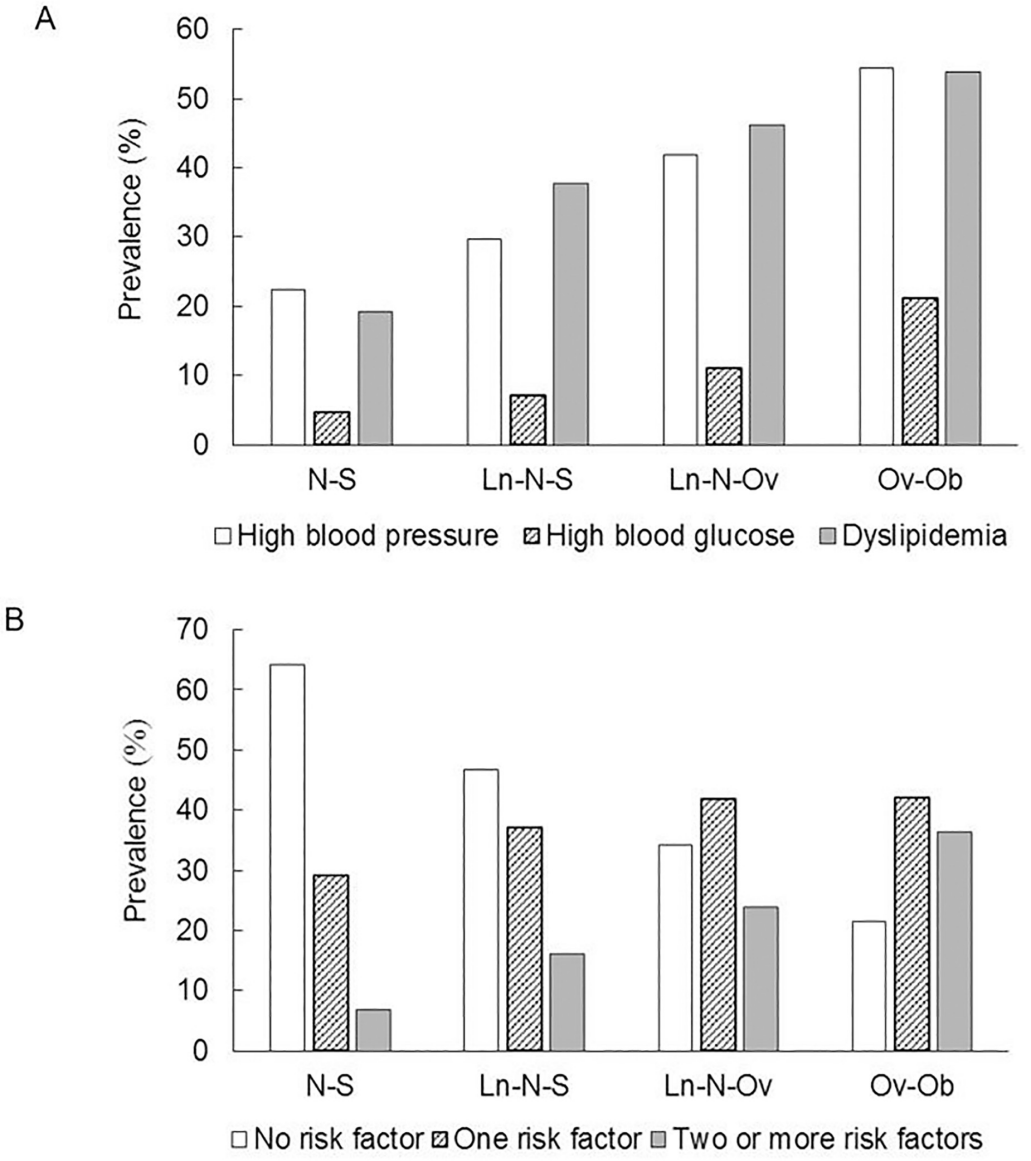
The prevalence of specific (A) and clustered (B) cardiovascular risk factors across BMI trajectory patterns. Abbreviations: N-S = Normal- Stable; Ln-N-S = Low normal-Normal- Stable; Ln-N-Ov = Low normal-Normal-Overweight; Ov-Ob = Overweight-Obese.

Across four trajectory patterns, the prevalence ranged from 22.4% to 54.5% for high blood pressure, from 4.8% to 21.1% for high blood glucose, from 19.2% to 53.8% for dyslipidemia, and ranged from 6.8% to 36.4% for having clustered cardiovascular risk factors (e.g., two or more risk factors). The prevalence was the lowest in those with N-S trajectory pattern and the highest in those with Ov-Ob pattern (Fig 2).

In model 1 (crude model), compared to participants with N-S trajectory patterns, those with other trajectory patterns had higher odds ratios of high blood pressure (1.4–4.1), blood glucose (1.5–5.2), dyslipidaemia (2.5–4.9), having any of the three risk factors (2.1–6.5) and having clustered risk factors (3.2–15.8). The Odds ratios remained significant after adjustment of socio-demographic factors in model 2 and further adjustment of lifestyle factors at the last follow-up in model 3 (Table 2).

**Table 2:** Association between body mass index trajectory pattern and cardiovascular risk factor in adulthood (age 26–80) (n = 2411).

	Odds ratio (95% confidence interval)^c^									
	N-S	Ln-N-S			Ln-N-Ov			Ov-Ob		
Cardiovascular risk factors		Model 1	Model 2	Model 3	Model 1	Model 2	Model 3	Model 1	Model 2	Model 3
High blood pressure	Ref	1.4 (1.1–1.8)	1.6 (1.3–2.1)	1.6 (1.3–2.1)	2.4 (1.9–3.2)	3.3 (2.5–4.4)	3.3 (2.5–4.3)	4.1 (2.8–6.0)	6.6 (4.5–9.8)	6.6 (4.5–9.8)
High blood glucose	Ref	1.5 (0.9–2.4)	1.7 (1.1–2.7)	1.7 (1.0–2.7)	2.4 (1.5–3.9)	3.3 (2.0–5.3)	3.2 (2.0–5.3)	5.2 (3.0–9.1)	8.8 (4.9–15.9)	9.1 (5.0–16.5)
Dyslipidaemia	Ref	2.5 (1.9–3.2)	2.6 (2.0–3.4)	2.6 (2.1–3.4)	3.5 (2.7–4.6))	4.0 (3.0–5.2)	4.0 (3.0–5.3)	4.9 (3.3–7.2)	5.8 (3.9–8.7)	5.9 (3.9–8.8)
Having any cardiovascular risk factors^a^	Ref	2.1 (1.6–2.4)	2.3 (1.8–2.8)	2.3 (1.8–2.8)	3.4 (2.7–4.3)	4.6 (3.5–5.9)	4.5 (3.5–5.8)	6.5 (4.3–9.9)	10.0 (6.5–15.4)	10.0 (6.4–15.4)
Number of cardiovascular risk factors^b^										
1	Ref	1.7 (1.4–2.1)	1.9 (1.5–2.4)	1.9 (1.5–2.4)	2.6 (2.0–3.4)	3.5 (2.6–4.5)	3.4 (2.6–4.5)	4.3 (2.7–6.8)	6.2 (3.9–10.0)	6.2 (3.9–9.9)
≥2	Ref	3.2 (2.2–4.5)	3.9 (2.7–5.6)	3.9 (2.6–5.5)	6.5 (4.4–9.5)	9.9 (6.6–14.8)	9.9 (6.6–14.8)	15.8 (9.3–27.0)	30.7 (17.5–54.0)	30.9 (17.6–54.4)

Abbreviations: N-S = Normal-Stable; Ln-N-S = Low normal-Normal-Stable; Ln-N-Ov = Low normal-Normal-Overweight; Ov-Ob = Overweight-Obese

^a^Having at least one of high blood pressure, high blood glucose or dyslipidaemia.

^b^Participants without cardiovascular risk factor were considered as the reference group.

^c^Model 1 was a crude model, model 2 was adjusted for socio-demographic factors (i.e., age, sex, living region and education), and model 3 was further adjusted for lifestyles (i.e., smoking, alcohol overconsumption, physical activity and unhealthy dietary pattern) at the last follow-up.

After stratified by age at last follow-up, compared with those having N-S pattern, those with Ln-N-S pattern, Ln-N-Ov pattern and Ov-Ob pattern had significantly higher odds of having any cardiovascular risk factors in all age group, with the odds ratio of 3.3, 6.9 and 14.0, respectively, in young adulthood, 1.8, 3.7 and 6.6, respectively, in midlife, and 3.1, 3.1 and 11.6, respectively, in late life. The results were similar after additional adjustment of socio-demographic factors in model 2 and further adjustment of lifestyle factors in model 3 (Table 3).

**Table 3:** The association between body mass index trajectory patterns and having any cardiovascular risk factors during different life periods.

Age at last follow-up (years)	No. of subjects	No. of cases	Odds ratio (95% confidence interval) ^a^									
			N-S	Ln-N-S			Ln-N-Ov			Ov-Ob		
				Model 1	Model 2	Model 3	Model 1	Model 2	Model 3	Model 1	Model 2	Model 3
Young adulthood (age 26–44)	318	119	Ref	3.3 (1.5–7.3)	4.0 (1.7–9.1)	4.2 (1.8–9.6)	6.9 (3.1–15.4)	9.2 (4.0–21.2)	8.7 (3.7–20.1)	14.0 (4.4–43.8)	19.2 (5.8–63.5)	19.0 (5.6–64.2)
Midlife (age 45–59)	1632	880	Ref	1.8 (1.4–2.6)	2.0 (1.5–2.6)	1.9 (1.5–2.5)	3.7 (2.7–4.9)	4.5 (3.3–6.1)	4.4 (3.2–6.0)	6.6 (4.1–10.8)	9.3 (5.6–15.4)	9.4 (5.7–15.4)
Late life (age ≥60)	461	286	Ref	3.1 (2.0–4.9)	3.1 (1.9–4.8)	3.1 (2.0–4.9)	3.1 (1.7–5.4)	2.9 (1.6–5.3)	3.1 (1.7–5.7)	11.6 (1.4–93.8)	10.8 (1.3–89.5)	10.8 (1.3–89.5)

Abbreviations: N-S = Normal-Stable; Ln-N-S = Low normal-Normal-Stable; Ln-N-Ov = Low normal-Normal-Overweight; Ov-Ob = Overweight-Obese

^a^Model 1 was a crude model, model 2 was adjusted for socio-demographic factors (i.e., age, sex, living region and education), and model 3 was further adjusted for lifestyles (i.e., smoking, alcohol overconsumption, physical activity and unhealthy dietary pattern) at the last follow-up.

The multiple imputation showed that the trajectory patterns from each of the five imputed datasets were similar with identical group percentage as that in the original dataset.

## Discussion

In this longitudinal population-based study, four distinct BMI trajectory patterns were identified over the life course: N-S, Ln-N-S, Ln-N-Ov and Ov-Ob. Compare to those with the N-S pattern, participants with Ln-N-S pattern, Ln-N-Ov pattern or Ov-Ob pattern had a higher future cardiovascular risk throughout adulthood, i.e., from young adulthood, midlife to late life, and the risk was highest in those with Ov-Ob pattern.

Since there are very few evidences on the trajectory of BMI over life course, therefore, we compare with other studies on the BMI pattern in specific life periods. We observed a continuous increase of BMI from age 6 to age 60 in all trajectories. These patterns from childhood to adulthood were similar to those reported from other studies on BMI trajectories [12,16, 23–24]. Moreover, a Canadian study reported less variability in the trajectory patterns during middle age [36], whereas we found distinct different patterns in midlife which was comparable with the Young Finns study [24]. Notably, our results were unique by showing the continuous change of BMI in late life (age 60–80). We found a decrease in BMI after age 60 in three trajectory patterns (i.e., N-S, Ln-N-S, Ln-N-Ov), whereas the BMI continued increasing until age 80 for the Ov-Ob trajectory pattern. These results were different from other studies which showed a stable BMI in late life [37,38]. However, these studies were lack of the trajectory of BMI prior to late life.

In addition, we linked the life-course trajectory pattern of BMI to several cardiovascular risk factors (e.g., high blood pressure, high blood glucose and dyslipidemia). In comparison with those having stable BMI over life course, participants with increasing BMI had higher cardiovascular risk from adulthood to late life. Although those with Ln-N-S pattern had normal BMI over life course, they still had higher cardiovascular risk than those with N-S pattern. The explanation might be the catch-up growth, characterized by a low BMI in the beginning of life and a steeper increase after that. This relationship between catch-up growth and cardiovascular risk in adulthood or late life was shown previously [39]. Furthermore, the group with Ov-Ob trajectory pattern had persistent overweight or obesity over life course. The long duration of obesity causes even higher risk of CVDs late in life [40], thus, it is easy to understand why this group of people had the highest cardiovascular risk. In addition, previous studies showed that the cardiovascular risk caused by childhood obesity could be eliminated when those obese children became non-obese adults [41,42]. However, we were unable to discover the group of people with overweight or obesity in childhood and normal BMI in adulthood.

Moreover, our study showed that the association between BMI trajectory patterns and cardiovascular risk factors were independent of the measurement time of cardiovascular risk factors. These findings suggested that the cardiovascular risk associated with unfavourable BMI change started early from young adulthood and continued into late life.

The major strength of this study is the longitudinal design with 20-year’s follow-up and seven waves of BMI measurements, which allowed us to predict the BMI trajectory from age 6 to age 80. Moreover, the study participants of CHNS were selected through a multi-stage sampling method and weighted sampling scheme, which made the study population a representative sample of Chinese population [25]. In regard to the random sample and the large number of participants, the findings from this study can be generalized to the whole China. However, the generalizability might be limited to the population from a different culture and background. The same protocol for data collection was used in all the waves making the data and measurements reliable with minimal internal variability. In addition, comprehensive measurements of cardiovascular risk factors provided the opportunity of assessing the subsequent cardiovascular risk of BMI trajectory patterns.

However, this study also has some limitations. Firstly, the missing BMI due to lost to follow-up or death across the six waves of follow-ups (21–55%) might affect the study results. The lost to follow-up was associated with younger age, living in an urban area, higher level of education and lower level of BMI at baseline compared with those followed in 2009 (S3 Table). However, the missing data might not significantly affect the main results since multiple imputation on the missing values showed similar trajectory patterns. Notably, death was not taken into account in the multiple imputation due to many missing data on death (52.8%), thus, the results from multiple imputation should be interpreted with caution. Secondly, the covariates (e.g., lifestyle factors) included in the analyses were from the last follow-up (2009) when the study outcomes were measured. This might bias the results if these lifestyle factors are the intermediate risk factors on the causal pathway between BMI and cardiovascular risk factors [43]. Futhermore, we didn’t take into account the change of these lifestyles factors during the follow-up period. Thirdly, we did not have any data on use of medications, especially antihypertensive, antidiabetic and lipid-lowering medications, that might underestimate the odds of cardiovascular risk factors. Finally, the LCGM with the best model fit was unable to discover the group of people with fluctuant BMI, e.g., gaining and losing weight (from overweight and obesity to normal), which indeed happens in reality. In regard to this, the caution is needed for the generalizability of our study findings.

Our study assessed the effect of life-course BMI change on cardiovascular risk factors later in life. The study findings identified the high-risk group of cardiovascular risk factors, which is considered as the ‘best buy’ approach for the prevention of CVDs. In this study population, BMI increased dramatically from childhood, young adulthood to midlife, and most of the overweight or obese children became obese adults. Those with increasing BMI over the life course had the highest cardiovascular risk later in life, and the risk seems to start early from young adulthood. These findings indicate the importance of early prevention of CVDs by controlling BMI within a normal range over life time and avoiding the rapid gain of BMI. Thus, a regular weight check and a weight control intervention (e.g., healthy diet and regular physical exercise) are needed to control the BMI within normal range across life course. Much more attention is needed for those vulnerable sub-groups with high risk of overweight and obesity (e.g., having a sedentary and unhealthy lifestyle).

In conclusion, four distinct BMI trajectory patterns were identified over the life course in the Chinese population. People with life-course stable BMI within normal range had the lowest cardiovascular risk whereas those with persistent overweight and obesity had the highest future cardiovascular risk. The cardiovascular risk associated with unfavourable BMI trajectory pattern started early in young adulthood.

## Supporting information

S1 File Description of latent class growth modelling (LCGM). (DOCX)

S1 Table The parameters for four trajectory classes from latent class growth modelling. (DOCX)

S2 Table Naming of the body mass index trajectory groups. (DOCX)

S3 Table Comparison of the baseline characteristics between participants followed in 2009 and those lost to follow-up. (DOCX)
