# Supplementary material for: Life-course trajectories of body mass index and subsequent cardiovascular risk among Chinese population
Source: PLoS One. 2019 Oct 10;14(10):e0223778. doi: 10.1371/journal.pone.0223778 (PMC6786833; doi:10.1371/journal.pone.0223778)
Supplement: S1 File — (DOCX) [file pone.0223778.s001.docx]

**S1 File. Description of latent class growth modelling (LCGM).**

LCGM analysis was used to identify distinct subgroups of individuals following a similar pattern of BMI change over time. Although each individual in CHNS had an unique BMI trajectory, the heterogeneity or the distribution of individual differences in change within the CHNS data was summarized by a finite set of unique polynomial functions each corresponding to a discrete trajectory pattern. LCGM model building was done based on the value of Bayesian Information Criterion (BIC), the significance of polynomial patterns, group membership probabilities, posterior probabilities and average posterior probabilities [35]. BIC value was used to compare competing models that include different numbers of trajectories or trajectories of various shapes (e.g., linear versus quadratic). BIC was recorded for each model, and lowest BIC value was used to find the optimal number of classes or trajectory patterns. The significance of polynomial terms was used to identify the shape of the classes or trajectory patterns [35]. LCGM also provided information regarding group membership probabilities, which indicated the aggregate size of each trajectory or the number of participants in a given trajectory. Preferably, each trajectory should have at least five percent of group membership probability [35].

The calculated posterior probabilities was used to assign each individual membership to the trajectory pattern/class that best matches participant's BMI change. A highest probability assignment rule was then used to assign each individual membership to the trajectory to which the participant held the highest posterior membership probability [35]. Following that, the average posterior probability of group membership for a trajectory was calculated, which represented the internal reliability for each trajectory. The calculation was done by averaging the posterior probabilities of individuals having been assigned group membership to a trajectory using the highest probability assignment rule [35]. Value of average posterior probabilities of group membership greater than 0.80 was taken into consideration to indicate that the modelled trajectories grouped individuals with similar patterns of change and discriminated between individuals with dissimilar patterns of change [35].

Specifically, model selection started with single trajectory, and more trajectory patterns were added if the model with the added groups had a better fit based on the criteria presented in S1 Table. During LCGM analysis, specification of the number of trajectories was done manually. The knowledge concerning the possible number and shape of the latent trajectories was identified from the existing literature in this arena. Three possible polynomial specifications were used to describe the shape of the longitudinal BMI trajectories as a function of age: a linear, and also quadratic and a cubic specification. Of these, successive polynomial models were tested between the trajectories, and the model with lowest BIC value (47650.84) was chosen as the best and final model. For model selection, linear or quadratic terms with significant coefficients were kept. The average posterior probability of each trajectory group exceeded or equaled to 0.80. The identified BMI trajectories differed in baseline values of BMI at the age of 6 years with increasing value of BMI over time.
