# Supplementary material for: Life-course trajectories of body mass index and subsequent cardiovascular risk among Chinese population
Source: PLoS One. 2019 Oct 10;14(10):e0223778. doi: 10.1371/journal.pone.0223778 (PMC6786833; doi:10.1371/journal.pone.0223778)
Supplement: S1 Table — (DOCX) [file pone.0223778.s002.docx]

**S1 Table. The parameters for four trajectory classes from latent class growth modelling.**

| Trajectory groups | Intercept (SE) | Linear term (SE) | Quadratic term (SE) | GMP (%) | Ave PP |
| --- | --- | --- | --- | --- | --- |
| Class 1 | 17.50355 (0.48135) | 0.09143 (0.02154) | -0.00080 (0.00023) | 22.4 | 0.86 |
| Class 2 | 13.39077 (0.15181) | 0.32287 (0.00796) | -0.00257 (0.00010) | 44.1 | 0.80 |
| Class 3 | 20.11880 (0.24321) | 0.19963 (0.00596) | - | 6.3 | 0.90 |
| Class 4 | 14.75260 (0.28741) | 0.34678 (0.01542) | -0.00237 (0.00019) | 27.2 | 0.82 |

GMP=Group membership probability; AvePP=Average posterior probability; SE=standard error
