# Supplementary material for: Life-course trajectories of body mass index and subsequent cardiovascular risk among Chinese population
Source: PLoS One. 2019 Oct 10;14(10):e0223778. doi: 10.1371/journal.pone.0223778 (PMC6786833; doi:10.1371/journal.pone.0223778)
Supplement: S2 Table — (DOCX) [file pone.0223778.s003.docx]

**S2 Table. Naming of the body mass index trajectory groups.**

| Trajectory groups | Body mass index status in different life period | | | | Unique name | Abbreviations |
| --- | --- | --- | --- | --- | --- | --- |
|  | Childhood  (age 6-17) | Adulthood  (age 18-44) | Midlife  (age 45-59) | Late life  (age ≥60) |  |  |
| Class 1 | Normal | Normal | Normal | Normal | Normal- Stable | N-S |
| Class 2 | Low normal | Normal | Normal | Normal | Low normal-Normal- Stable | Ln-N-S |
| Class 3 | Overweight | Overweight | Obese | Obese | Overweight-Obese | Ov-Ob |
| Class 4 | Low normal | Normal | Overweight | Overweight | Low normal-Normal-Overweight | Ln-N-Ov |
