# Supplementary material for: Life-course trajectories of body mass index and subsequent cardiovascular risk among Chinese population
Source: PLoS One. 2019 Oct 10;14(10):e0223778. doi: 10.1371/journal.pone.0223778 (PMC6786833; doi:10.1371/journal.pone.0223778)
Supplement: S3 Table — (DOCX) [file pone.0223778.s004.docx]

**S3 Table. Comparison of the baseline characteristics between participants followed in 2009 and those lost to follow-up.**

| Baseline characteristics | Participants followed in 2009 (*N*=2411) | Those lost to follow-up (*N*=2865) | *p* |
| --- | --- | --- | --- |
| Age, years, mean (SD) | 33.4 (7.0) | 29.0 (9.4) | < 0.001 |
| Living region, n (%) |  |  |  |
| Urban | 591 (24.5) | 1070 (37.3) | < 0.001 |
| Rural | 1820 (75.5) | 1795 (62.7) |  |
| Sex, n (%) |  |  |  |
| Male | 1125 (46.7) | 1384 (48.3) | 0.234 |
| Female | 1286 (53.3) | 1181 (51.7) |  |
| Education, n (%) |  |  |  |
| No formal school | 765 (32.0) | 589 (21.2) | < 0.001 |
| Primary school | 630 (26.3) | 573 (20.6) |  |
| Middle school and above | 997 (41.7) | 1619 (58.2) |  |
| Body mass index, kg/m^2^, mean (SD) | 21.6 (2.4) | 21.0 (2.8) | < 0.001 |
| Body mass index status, n (%) |  |  |  |
| Normal | 2027 (84.1) | 2493 (87.0) | 0.009 |
| Overweight | 352 (14.6) | 337 (11.8) |  |
| Obese | 32 (1.3) | 35 (1.2) |  |
